# Individualized medicine and the microbiome in reproductive tract

**DOI:** 10.3389/fphys.2015.00097

**Published:** 2015-04-01

**Authors:** Andrea G. Braundmeier, Katherine M. Lenz, Kristin S. Inman, Nicholas Chia, Patricio Jeraldo, Marina R. S. Walther-António, Margret E. Berg Miller, Fang Yang, Douglas J. Creedon, Heidi Nelson, Bryan A. White

**Affiliations:** ^1^Department of Medical Microbiology, Immunology and Cell Biology, Department of Obstetrics and Gynecology, Southern Illinois University School of MedicineSpringfield, IL, USA; ^2^Department of Cancer Biology, Mayo Clinic College of MedicineJacksonville, FL, USA; ^3^The Center for Individualized Medicine, Mayo ClinicRochester, MN, USA; ^4^Division of Nutritional Sciences, University of IllinoisUrbana, IL, USA; ^5^Biomedical Engineering and Physiology, Mayo CollegeRochester, MN, USA; ^6^Carl R. Woese Institute for Genomic Biology, University of IllinoisUrbana, IL, USA; ^7^Department of Surgery, Mayo ClinicRochester, MN, USA; ^8^Department of Obstetrics and Gynecology, Mayo ClinicRochester, MN, USA

**Keywords:** individualized medicine, microbiome, reproductive tract infections, Pregnancy, biomarkers

## Abstract

Humans have evolved along with the millions of microorganisms that populate their bodies. These microbes (10^14^) outnumber human cells by 10 to 1 and account for 3 × 10^6^ genes, more than ten times the 25,000 human genes. This microbial metagenome acts as our “other genome” and like our own genes, is unique to the individual. Recent international efforts such as the Human Microbiome Project (HMP) and the MetaHIT Project have helped catalog these microbial genomes using culture-independent, high-throughput, next-generation sequencing. This manuscript will describe recent efforts to define microbial diversity in the female reproductive tract because of the impact that microbial function has on reproductive efficiency. In this review, we will discuss current evidence that microbial communities are critical for maintaining reproductive health and how perturbations of microbial community structures can impact reproductive health from the aspect of infection, reproductive cyclicity, pregnancy, and disease states. Investigations of the human microbiome are propelling interventional strategies from treating medical populations to treating individual patients. In particular, we highlight how understanding and defining microbial community structures in different disease and physiological states have lead to the discovery of biomarkers and, more importantly, the development and implementation of microbial intervention strategies (probiotics) into modern day medicine. Finally this review will conclude with a literature summary of the effectiveness of microbial intervention strategies that have been implemented in animal and human models of disease and the potential for integrating these microbial intervention strategies into standard clinical practice.

## Understanding the human microbiome

As humans evolved, so did the millions of microorganisms that populate the human body. These microbes (10^14^) outnumber human cells 10 to one and account for 3 × 10^6^ genes, more than 10 times the 25,000 genes comprising the human genome. This microbial metagenome acts as an “extended human genome,” and, like our own genes, is highly specific to the individual. Recent international efforts such as the Human Microbiome Project (HMP) and the MetaHIT Project have helped catalog these microbial genomes using culture-independent, high-throughput, next-generation sequencing (Cai et al., 2010; Human Microbiome Jumpstart Reference Strains Consortium et al., 2010; Lozupone et al., 2012; Human Microbiome Project Consortium, 2012a,b). They have shown that, out of 55 bacterial divisions, the metagenome is dominated by only a few bacterial phyla (Firmicutes, Bacteriodetes, Actinobacteria, and Proteobacteria) (Ley et al., 2007). The microbiome performs both digestive and metabolic functions, and is responsible for 40% of the host's energy intake (Round and Mazmanian, 2009). Another important role for the microbiome is development of the host's immune system, as mice lacking gut microbes are defective in immune cell maturation and response (Round and Mazmanian, 2009; Chung et al., 2012). Importantly, perturbations in the microbiome can lead to both metabolic and immune diseases, highlighting the importance of understanding the “healthy” human microbiome and its interaction with the host. Symbiosis between host and the gut microbiome plays an important role in maintaining a physiological homeostasis. Supporting this, an individual's microbiome phylotype is specific and stable, suggesting that each host has a unique relationship with its microbiome (Costello et al., 2009). In other words, there is an individualized core microbiome responsible for maintaining a healthy state, and any significant deviation from that core may affect an individual's risk for disease (Sartor, 2004; Ley et al., 2006; Manichanh et al., 2008).

Historically, identification of individual microbes has been difficult because of the inability to culture many bacteria *in vitro*. With the genomic era came the development of culture-independent technologies (genome sequencing) that allowed for genomic sequencing of 16S ribosomal genes that accurately identified and allowed for classification of millions of bacterial species (Tringe et al., 2005; Mardis, 2008).

The precepts of the HMP provide a foundation and model for advancing the tools, approaches, databases and fundamental contributions of microbiome research to health and disease far beyond Koch's Postulates into the realm of individualized medicine. The application of these approaches, and the resulting concepts, span far beyond the microbiomes of sympatric microbial relationships found in humans to those in food animals, non-human primates and free living communities. Our understanding of the human microbiome has altered the future landscape of our approach to health and disease. We now consider the individual as a superorganism, combining our unique human genome with the microbiome, including an assessment of microbial population structure with an assessment of how the microbial community functions. In light of this new genomic revolution, we will review the microbiomes of the female reproductive tract, focusing on the role of the vaginal and placental microbiomes, diseases that result from disruption of these microbiomes and therapies based on manipulation of these microbiomes (Table 1).

**Table 1:** **Summary of literature on microbial dynamics and reproductive physiology**.

**Reproductive State/disorder**	**Effect on microbiome**	**References**
Menstruation	Altered vaginal microbiome stability, but no change in microbial function	Gajer et al., 2012
Placental function	Modulation of placental function and metabolism	Aagaard et al., 2014
Preterm birth (PTB)	Altered microbial diversity, but not richness; Associated with *Lactobacillus iners*	Brocklehurst et al., 2013 Barbonetti et al., 2011
Pregnancy	Reduction in microbial richness and diversity; Induced microbial stability	Dechen et al., 2010 Barbonetti et al., 2011; Aagaard et al., 2012
Vaginal parturition	Colonization of fetal microbiome	Dominguez et al., 2003; Huurre et al., 2008; Groeger et al., 2013; Tojo et al., 2014
Bacterial vaginosis (BV)	Altered vaginal bacterial colonization; Associated with PTB, STDs, and UTIs	Roger et al., 2010; Aaltonen et al., 2011; Brotman, 2011
Pelvic inflammatory disease (PID)	Ascension of pathogens from the lower to upper reproductive tract; Pathogens connected to PID also associated with BV	van Oostrum et al., 2013
STDs/STIs	Associated with BV; Decreased vaginal microbiome-vaginal cavity immune system relationship	Roger et al., 2010; Jaiyeoba et al., 2011; Taylor et al., 2013 Klebanoff and Coombs, 1991; Hill and Anderson, 1992; Brotman et al., 2007, 2010; Mirmonsef et al., 2011
Cancers of the reproductive tract	Shift from protective to harmful bacteria; Associated with viral infections (HIV, HPV); Link between BV and precancerous lesions/malignancy progression of cervical cancer	Nicol et al., 2005 Martin et al., 1999; Brotman et al., 2010 Barrington et al., 1997; Parkin, 2006; Denslow et al., 2011
Ovarian stimulation for IVF	Altered vaginal microbiome, but no change in diversity of microbial species	Hyman et al., 2013
Estrogen circulation	Estrabolome metabolizing functions	Cole et al., 1985; Gadelle et al., 1985; Dabek et al., 2008; Hou et al., 2013; Mandar, 2013

## Reproductive tract microbiomes

### Anatomical features of the reproductive system for pathogen resistance

The female reproductive tract is composed of (anterior to posterior): the ovaries, fallopian tubes, uterus, cervix, and vagina. The anterior structures of the reproductive system represent a bacterial naive environment that ensures efficient gamete production and transport, fertilization of the ovum, embryonic development and implantation due to the absence of inflammatory immune responses. As the outer orifice to the reproductive tract, the vagina serves as the first line of defense to protect against entry of harmful bacteria and other pathogens into the immunologically privileged sites of the uterus and fallopian tubes. The microbial communities of the vagina serve as the “guard dogs,” along with the acidic pH, to regulate microbial growth and transmission. Anterior to the vagina lies the cervix, which serves as a physical barrier for pathogen entry to the uterus and fallopian tubes. Both the vagina and cervix work together, albeit through different mechanisms, to protect and maintain reproductive health. The cervical rings or folds create an obstacle course for both pathogens and sperm cells alike to traverse, while the hormonally regulated secretions of the cervix can create either a viscous (post-ovulation) or watery (pre-ovulatory) lubrication for flushing microorganisms out of the vagina during copulation (Yarbrough et al., 2014). Infections of the upper reproductive tract occur when microbial communities of the vagina are altered to permit the overgrowth of harmful microorganisms that then overwhelm the cervical barrier and breach the immune protection and negatively impact the commensal bacteria of the upper reproductive system. This breach results in the pathogenesis of reproductive disorders including preterm birth (PTB), pelvic inflammation, sexual transmitted diseases, gynecological cancers and other diseases. Understanding and more importantly, maintaining a healthy vaginal microbial environment is critical to preventing many reproductive disorders associated with many pathogens.

### The vaginal microbiome

The human vaginal microbiome undergoes continuous evolution as the host encounters and responds to a number of environmental and physiological disruptions. Application of genomic technologies (mentioned above) to the vaginal ecosystem has identified five distinctive microbial communities, the specific proportions of which are significantly shaped by a woman's race/ethnicity (Ravel et al., 2011) and personal hygiene habits (Ness et al., 2002). *Lactobacillus* is the dominant family of bacterial species (*L. crispatus, L. iners, L. gasseri, and L. jensenii*) comprising four out of the five major vaginal microbial communities, with the final microbial community composed of a variety of strict and facultative anaerobes (Ravel et al., 2011). In addition, identification of species composition is valuable but more important is the functionality of microbial species within these unique communities. *Lactobacillus* species function to protect the reproductive tract utilizing several mechanisms, primarily through production of lactic acid, which lowers the pH of the vaginal environment, and through activation innate immune responses (Kim et al., 2006; Fichorova et al., 2011). Mechanisms employed by non-*lactobacillus*-dominant communities to regulate the vaginal ecosystem are relatively unexplored but would greatly enhance the understanding of how a “healthy” vaginal environment is maintained in the presence of diverse environmental and physiological agitations.

#### Hormonal regulation

Reproductive cyclicity is defined as repeated opportunities for conception, implantation and pregnancy. Cyclicity is regulated by ovarian hormones and involves dynamic changes in the ovary and also endometrial remodeling. Menstruation is a physiological event that initiates uterine endometrial remodeling required for pregnancy in humans and some non-human primates. The fall of ovarian hormones estrogen and progesterone triggers menstruation but is also associated with changes in vaginal microbial communities. Very few studies have investigated the regulation of microbial communities by ovarian hormones (Hickey et al., 2013) and only one study has reported changes in microbial community stability throughout the menstrual cycle (Gajer et al., 2012). The Gajer study looked at the vaginal microbiome stability over a period of 16 weeks in 32 reproductive aged women. This study concluded that the Jensen-Shannon divergence rate of change (i.e., a measure of the bacterial deviation from constancy) was lowest when estradiol levels were the highest, indicating an inverse relationship between constancy and estradiol levels. The greatest divergence rate of change was found at the onset of menstruation, gradually declined as estradiol levels rose and then quickly rose during the late secretory stage (decline of estradiol levels) of the menstrual cycle. Although the authors found greater community stability under estrogen dominance this did not coincide with an increase of community diversity. Additionally, the change in community stability did not lead to fluctuations in microbial function (maintenance of vaginal pH and lactic acid production), indicating that there can be redundant functions in microbial species within a community. Individuals who have lost this redundancy may be those more susceptible to reproductive disorders associated with infertility and reproductive disease states.

### Placental and amniotic fluid microbiomes

For several decades we have known that bacteria are found in what is considered to be “sterile” environments such as the uterus and fetal tissues (Harris and Brown, 1927). More recent investigations have supported this original observation that the placenta and amniotic fluid (AF) surrounding the fetus during pregnancy are not “sterile” environments and that the presence of bacterial species has an impact on gestation and parturition (Satokari et al., 2009; Fardini et al., 2010; DiGiulio, 2012; Mendz et al., 2013; Aagaard et al., 2014; Doyle et al., 2014). A recent review of investigations that characterized bacteria found in the AF has elucidated that while the presence of bacteria in AF is associated with adverse obstetrical outcomes, bacteria are still present in the AF of women with “healthy” term pregnancies (Payne and Bayatibojakhi, 2014). Interestingly, it has been reported that the placenta contains similar bacterial species to that found in the oral cavity (Douvier et al., 1999). However, this study was limited as it utilized only placental tissue from patients with known chorioamnionitis (infection of the chorion and amnion layer of the placenta) and also relied on culture based detection methods to identify bacterial species. With the emergence of powerful DNA sequencing techniques, it has now been reported that even non-infectious “healthy” placental tissue harbors a number of microbial species establishing that placental tissue has a microbial signature similar to the oral cavity (Satokari et al., 2009; Aagaard et al., 2014; Antony et al., 2014; Doyle et al., 2014). Hence transmission of bacterial species from the oral cavity to the AF and placenta can be benign or result in adverse pregnancy outcomes (Hill, 1998; Han et al., 2004, 2006, 2009) (to be discussed later). In addition, transmission of certain bacterial species, Streptococcus, Escherichia coli, Shigells, Proteus, Enterobactera and Candida from the gastrointestinal (GI) tract to reproductive tissues (vagina, endometrium, and placenta) also negatively impact pregnancy (Dechen et al., 2010) but have been shown to have important roles in amino acid metabolism and positively impact sperm transport and fertility (Barbonetti et al., 2011). Similarly, there are immunologically beneficial bacterial species from the gastrointestinal (GI) tract to the placenta, specifically bifidobacteria or lactobacilli, have been shown to benefit fetal development through early programming of the naïve fetal immune system and protection of the fetus from activated maternal immune cells (Satokari et al., 2009). Hence the transmission of bacterial species from other mucosal tissues to the placenta and amniotic fluid throughout gestation has both symbiotic and pathogenic outcomes.

### Microbial influences on reproductive function (symbiosis to pathogenesis)

#### Symbiotic relationships

#### Pregnancy

Pregnancy is considered a “healthy” parasite-host physiological state, which is supported by elevated levels of both estrogen and progesterone. The presence of a growing fetus (parasite), elevated ovarian hormone levels and placental factors induces dynamic changes in the mother's (host) microbial communities. Recently, a comprehensive study investigating microbial shifts throughout pregnancy reported that as early as 18–24 gestational weeks there was alteration of microbial richness in the vaginal microbiome (Aagaard et al., 2012). This study also reported that while gestational timing and vaginal location were important in determining microbial community structure that overall throughout the entire pregnancy there were reductions in both microbial richness and diversity throughout all stages of pregnancy when compared to non-pregnant women (Aagaard et al., 2012). Further, investigation on the influence of pregnancy on specific bacterial taxonomy expression, showed that pregnancy was highly associated with the expression of *Lactobacillus, Bifidobacteriaceae and Streptococcaceae* operational taxonomic units. More recent data has confirmed that pregnancy induces microbial stability within the vaginal cavity dominated by *Lactobacillus* species (Petricevic et al., 2014; Walther-Antonio et al., 2014). Enhanced expression of members within these particular bacterial families may function to improve immune system function (Groeger et al., 2013) for protection of the fetus from the maternal immune response and to protect the uterine environment from pathogen invasion (Fichorova et al., 2011).

The placenta is a unique organ in that it is only present during pregnancy but is vital for the transfer of nutrients and other factors from maternal to fetal circulation to support fetal development. Thus it is not surprising that bacterial species that colonize the placental tissues have a unique metabolic capacity unlike that of other mucosal tissues. Using a metagenomic approach, Aagaard et al, was able to identify the metabolic profile of bacterial taxonomy found within the placenta (Aagaard et al., 2014). This study reported that the placenta had a higher abundance of bacterial gene profiles supporting metabolism of cofactors and vitamins than other observed tissues (stool, tongue, posterior vagina, etc.). They also observed a decreased abundance of bacterial gene profiles associated with carbohydrate and amino acid metabolism. Accordingly the relative abundance of metabolic pathways was influenced by both gestational age and also history of previous maternal infections. Collectively this study demonstrated that the bacteria colonizing placental tissues modulates overall placental function and may be indicative of placental and maternal health.

#### Fetal inoculation

As introduced earlier in this review, there is evidence that supports the benefit of bacterial transmission from the mother to the fetus during gestation. One major advantage of maternal transmission of bacterial strains to the fetus is to populate “naïve” fetal tissues, such as the gastrointestinal and immune systems, with beneficial bacteria that would aid fetal metabolism and confer immune protection upon birth. Although it has been highly reported that transmission of positive bacterial strains occurs during the descent of the fetus through the vaginal canal (as discussed below) there is also transmission of bacterial strains through placental hematogenous transmission (Fardini et al., 2010). Transmission of commensal intestinal bacteria, mainly *Bifidobacterium* and *Lactobacillus rhamnosus*, was found to be present in the majority of placental tissues examined, regardless of mode of delivery (Satokari et al., 2009). The identification of these particular bacterial species is highly significant because of the importance of these strains in modulating immune health (reviewed in Tojo et al., 2014).

Evidence suggests an additional role for the mother's vaginal microbial community, in colonizing the fetal microbiome during parturition. Fetal descent into the vaginal canal during parturition creates an intimate contact between fetal orifices (oral and rectal) and the maternal vaginal microbial communities. The theory of maternal colonization of the fetal microbiome has been validated through investigations comparing infant microbiome signatures of different delivery methods (Dominguez et al., 2003; Huurre et al., 2008) and gestational age at delivery (Vassallo and Walker, 2008; Fanca-Berthon et al., 2010). These studies found that infants born via vaginal delivery had “healthy” postnatal microbial communities similar to their mother's microbial communities. An example of postnatal inoculation is the increased levels of *Bifidobacterium* and *Lactobacillus* species found in fetal stool samples collected at different timepoints after birth (Makino et al., 2013), indicating further enrichment of the fetal GI microbiome from that which occurred in utero. Furthermore, infants born via vaginal delivery that were breastfed had gut microbiome profiles that favored greater immunological protection compared with children born via cesarean section (Gronlund et al., 2007; Roger et al., 2010; Aaltonen et al., 2011; Decker et al., 2011; Cabrera-Rubio et al., 2012; Guarino et al., 2012). Promoting these additional positive effects of vaginal delivery and breastfeeding may mold future practices for obstetrical care in the United States to reduce the rate of elective cesarean sections.

#### Pathogenic relationships

#### Bacterial vaginosis

Microbial community composition and richness is always subjected to a state of “normal” fluctuation. When the degree of fluctuation becomes too severe, the microbial relationships of these communities shift from symbiotic to pathogenic (disease). Bacterial Vaginosis is the condition of abnormal bacterial colonization of the vaginal cavity and has been associated with other pathological conditions such as infertility, PTB, sexual transmitted diseases, and urinary tract infections, among others (Quan, 2010; Brotman, 2011; Dielubanza and Schaeffer, 2011). Investigation into metabolic markers of BV has linked different disease symptoms (odor, epithelial integrity, discharge) with specific bacterial species (Yeoman et al., 2013). Utilization of 16S rDNA sequencing technology indicated that bacterial community richness and diversity of strictly anaerobic bacteria was increased in women with BV (Fredricks et al., 2005; Yeoman et al., 2013). However the same anaerobic species (Gardnerella, Atopobium, Mobiluncus and Prevotella) found in women with symptomatic BV are also similar to a bacterial community state found in women with asymptomatic healthy women (Ravel et al., 2011; Yeoman et al., 2013). The diversity of the vaginal microbiome among each individual complicates the ability to determine a bacterial “signature” of BV from that of the temporal changes in the vaginal microbiome due to reproductive cyclicity, personal hygiene or other numerous variables. This temporal fluctuation of the vaginal microbiome makes it difficult to identify bacterial species that are causative for BV or the invasion of opportunistic bacterial species that colonize and populate the vaginal microbiome during environmental changes of the vaginal cavity. The difficulty in identification of a causative pathogen for BV also increases the difficulty of developing effective treatments for BV.

Treatment protocols for BV include antibiotic therapy either with oral tablets or vaginal creams (Centers for Disease Control and Prevention et al., 2006). Additionally, the utilization of combo or progestin only oral contraception drugs (OCPs) has shown a protective effect to development of BV through hormonal stabilization of microbial communities in the vaginal canal (Rifkin et al., 2009). However, reoccurrence of disease within 1 year following the cessation of treatment is high (Bradshaw et al., 2006). The current medical challenges associated with treating BV are: (1) diagnosis in patients (especially those who are asymptomatic) (Ma et al., 2012; Drell et al., 2013), (2) treatment of disease (anti-microbial vs. probiotics) (reviewed in Donders, 2010; Brocklehurst et al., 2013) and (3) etiology of disease (fulfilling Koch's postulates of disease) (Fredericks and Relman, 1996). Thus, even centuries since its discovery, BV still remains a nuisance for clinicians worldwide and a precursor for the development of other health conditions.

#### Gynecological and obstetrical complications

Pregnancy-associated vaginal microbiome profiles can be utilized as screening tools to assess obstetrical and perinatal complications prior to symptomatic presentation. Multiple studies have investigated the correlation between PTB and the diversity and richness of the microbial communities within the vaginal canal. Using a culture- independent approach and chain terminator sequencing technology to analyze the vaginal microbiome of patients at risk for a subsequent pre-term birth, the authors found that both Caucasian and African American women with PTB had altered microbial diversity but not richness compared with the term gestational counterparts (Hyman et al., 2013). Investigations on the diversity of Lactobacillus species during pregnancy indicated that expression of *Lactobacillus iners* was highly associated with PTB (Petricevic et al., 2014). Additionally, it has been shown that women with bacterial vaginosis (BV), an abnormal bacterial colonization of the vaginal cavity, are at an increased risk for PTB, early pregnancy loss (van Oostrum et al., 2013) and failure of IVF therapy (Hyman et al., 2012). These data suggest that using more advanced “next-generation” sequencing to profile pregnancy-associated vaginal microbiome may help identify patients at high risk for PTB.

#### Pelvic inflammatory disease and sexual transmitted diseases/infections

Pelvic inflammatory disease (PID) is defined as infection and inflammation of the female genital tract. The pathobiology of this disease is due to the ascension of pathogens from the lower to the upper reproductive tract which can result in serious and long term negative implications for reproductive function (infertility, salpingectomy and/or hysterectomy). Many of the pathogens implicated in the etiology of PID are associated with BV (Taylor et al., 2013) but to date there is a lack of substantial evidence that demonstrates a causal relationship between particular organisms and the development of PID. Further evaluation of the microbiota in women with PID could elucidate specific organisms that can be utilized as a screening tool to identify women at risk for PID prior to onset of clinical systems. The current recommendations for treatment of PID are containment of pathogens in the genital tract and utilization of broad-spectrum antibiotics that adequately cover the associated species of microorganisms (Jaiyeoba et al., 2011).

Sexual transmitted disease (STD) and sexually transmitted infection (STI) acquisition are additional health concerns that are affiliated with common microorganisms, similar to BV (Brotman, 2011). Woman with reoccurring BV are also at a significantly elevated risk for many types of STD's or STI's including HIV (Kaul et al., 2008), gonococcal, chlamydial and trichomonal infections (Brotman et al., 2010). The association of BV occurrence with contraction of other STD's and STI's is due to a variety of factors. Patients with a high recurrence of BV are likely to also be infected with STDs due to sexual behavioral choices that can influence the vaginal microbiome such as participation in oral sex and lack of physical contraceptive utilization (Brotman et al., 2007). Additionally, the correlation between BV and STD/STI's may be reflective of the relationship between the vaginal microbiome and the host immunity of the vaginal cavity. Immune protection of the vaginal cavity is created by certain microorganisms (mainly *lactobacillus* genus) that: (1) neutralize and prohibit the colonization of harmful pathogens (bacterial and viral) and (2) activate the innate immunity of the lower genital tract (Mirmonsef et al., 2011). Lactobacilli production of high levels of hydrogen peroxide (H_2_O_2_) are toxic to HIV viral replication and inhibit CD4+ T cell activation therefore, decreasing HIV transmission (Klebanoff and Coombs, 1991; Hill and Anderson, 1992). Although communication between microbial communities and the immune system can be beneficial for protecting common pathogen entry niches, a disruption of the vaginal cavity's immune system could potentiate the development of reproductive diseases/infections and increase the severity of infection (Martin et al., 1999; Sha et al., 2005) which can predispose the vaginal mucosal membrane for the development of certain types of cancer (reviewed in Nicol et al., 2005).

#### Cancers of the reproductive tract

A disruption of the vaginal microbial communities can lead to decreased immune protection, thereby increasing susceptibility of the epithelial lining of reproductive tissues to viral infections. The shift of microbial communities from those that are protective (*Lactobacillus* genus) to those that are harmful anaerobic bacteria (*Gardenerella vaginalis, Atopobium vaginae, Mobiluncus* and *Prevotella species*), as occurs with BV, can damage the epithelial layer of reproductive tissues making it penetrable to viral entry, especially those viruses that are sexually transmitted (HIV and HPV) (Denslow et al., 2011; Mirmonsef et al., 2011). Importantly, infection with HIV or HPV is attributed to the development of several cancers including cervical cancer, Kaposi sarcoma, Non-Hodgkin lymphoma, ano-genital cancer, and cancers of the mouth and oropharynx (Parkin, 2006). Similarly, several studies have shown a linkage between the occurrence of BV and the development of precancerous lesions and malignancy progression in patients diagnosed with cervical cancer (Guijon et al., 1992; Platz-Christensen et al., 1994; Barrington et al., 1997). Furthermore, a recent meta-analysis reported a clear association between BV and cervical cancer (Gillet et al., 2012). These studies provide support for the importance of maintaining a “normal” vaginal ecosystem to avoid development of malignant pathology.

#### Infertility and assisted reproductive technology

Detailed research is lacking on the association between reproductive tract microbiomes (vaginal or uterine) and infertility rates or the successfulness of Assisted Reproductive Technologies (ART). It is known that inoculation of the uterine cavity with harmful bacteria by the embryo transfer catheter adversely effects positive pregnancy outcome (Egbase et al., 1996, 1999; Fanchin et al., 1998). Additionally, women with STD's or high reoccurring rates of BV also have an increased incidence of failed *in vitro* fertilization (IVF) embryo transfer (ET) attempts (Witkin et al., 1995; Liversedge et al., 1999). In contrast, while intercourse among heterosexual partners may influence the urogenital microbiomes of each individual the penile microbiome has not been shown to be associated with fertility status in males (Hou et al., 2013; Mandar, 2013). Together these data suggest that successful embryonic implantation can be impeded by the presence of potentially pathogenic bacterial species.

It is well documented that the vaginal microbiome is highly influenced by the secretion patterns of ovarian hormones (Ravel et al., 2011). Recently, the composition and diversity of the vaginal microbiome was analyzed in patients undergoing different ovarian stimulation protocols for IVF and correlated to clinical outcome (Hyman et al., 2012). The authors reported that ovarian stimulation did result in alterations of the vaginal microbiome but the diversity of the microbial species was not different among the different protocols tested. Importantly, microbiome composition at the time of embryo transfer was found to correlate with a successful IVF outcome as, patients for whom IVF resulted in a live birth had significant differences in their vaginal microbiomes compared to women who were unsuccessful at achieving pregnancy. Determining the mechanism by which the vaginal microbiome may influence ART outcomes would be highly beneficial for guiding clinical recommendations on which ART should be implemented, if any, to patients seeking infertility treatment.

#### Estrabolome

While the microbial community profiles of reproductive tissues are distinct from those reported in the gastrointestinal (GI) tract, it is important to mention that the function of reproductive organs is highly regulated by the estrogen metabolizing functions of bacterial species contained within the GI. It has long been known that bacterial species found in the GI tract possess enzymatic activity, which is essential for processing estrogens to be released back into circulation or conjugation of estrogens to be excreted through urine or fecal production (Cole et al., 1985; Gadelle et al., 1985; Dabek et al., 2008). This process is known as enterohepatic circulation (Adlercreutz et al., 1979) and the bacterial species regulating this process are collectively known as the estrabolome (reviewed in Plottel and Blaser, 2011). Alteration of the estrabolome can result in either increased or decreased peripheral circulating levels of estrogens, which can affect multiple reproductive functions including (ovarian and endometrial cyclicity, pregnancy, infertility (Obst and Seamark, 1975; Gautray et al., 1981; Filicori et al., 1991), infection (Luthje et al., 2014) and malignancy (Kabat et al., 1997; Gupta et al., 1998). This indicates the complexity of the regulation of physiological systems by microbial communities in different regions of the human body and stresses the importance of maintaining an overall “healthy” microbiome to achieve overall health.

### Individualized medicine and the human microbiome

#### Therapeutics

Microbiome-associated diseases are considered preventable or treatable in the individualized medicine paradigm. Potential means to maintain or restore the healthy state of a microbiome include use of probiotics or prebiotics, implementing a change in diet and use of unique gut- or reproductive-tract derived commensals as treatment options to generate a balanced microbiome. Mucosal surfaces, such as those of the reproductive and digestive tract, are important portals of entry for diverse environmental agents. Physical mucosal barriers and the mucosal immune system play critical defensive roles by limiting tissue damage from harmful exposures to foreign substances, including bacteria and antigens (reviewed in Kaushic, 2009). Mucosal interfaces are, not surprisingly, rich with immune cells and are highly regulated to maintain a balance between absorption of beneficial nutrients and protection against harmful organisms and antigen (Bradshaw et al., 2006; Hickey et al., 2011). Perturbations of this system are implicated in several common and chronic diseases, including inflammatory bowel disease (reactions to commensal organisms), celiac disease and food allergies (reaction to gluten and specific foods), *Helicobacter* related gastritis (peptic ulcer disease, gastric cancers related to *Helicobacter*), allergic disorders (associated with reaction to foods), aero-antigens, and fungi. If the mucosal environment represents a risk for contraction of local and distant disease, it also follows that it might serve as a target site for reducing risk through focused efforts to prevent or reverse harmful exposures. There is emerging interest in considering the beneficial effects of commensal organisms, probiotics, and prebiotics, fecal transplants and mucosal immune system vaccination strategies. However for this to be successful, we need to ascertain whether the compositional changes in the microbiome are consequences or driving forces of the different diseases, and finally, if different types of microbial therapeutics (individualized medicine) can alter the development of different diseases.

#### Probiotics

Probiotic therapy describes the ingestion of living organisms, or “good bacteria.” It is thought that probiotic therapy enhances nutrient uptake and immune health, and that this could alleviate reproductive disorders and reduce the occurrence of premature birth. While no direct evidence exists linking probiotic use with a reduction of reproductive complications, there is an abundance of evidence supporting probiotic utilization for improvement of immune, gut barrier, placental, and cardiovascular functions (reviewed in Reid et al., 2013). Dietary supplementation with certain probiotic formulations has shown to reduce inflammatory responses in pregnant women by counteracting the shift in microbial populations that can be inflammatory during late pregnancy (Vitali et al., 2012). These data suggest that although modulation of the vaginal and gastrointestinal microbiomes may not directly target the uterine microbiome, pregnancy outcome may be positively impacted through probiotic use through overall enhancement of maternal health.

Probiotic therapy plays a role in treatment of urogenital diseases as well (reviewed in Reid et al., 2004). Probiotic supplementation has been shown in pilot studies to be an equally (or more) effective, short term, treatment compared to standard clinical antibiotic treatments in patients with vaginitis and BV (Donders et al., 2010; Ling et al., 2013). Further analysis of one pilot study that utilized probiotics in patients with BV revealed that probiotics do not eliminate harmful taxonomy, as did antibiotics, but rather suppressed the overgrowth of bacterial strains associated with BV thus possibly leading to its enhanced effectiveness (Ling et al., 2013). Additionally, it has been reported that inoculation of the uterine microbiome with *Lactobacillus crispatus* at the time of embryo transfer can increase implantation rates and reduce colonization of pathogenic microorganisms (Sirota et al., 2014). To date there is limited evidence for the beneficial effects of probiotic therapy, strain composition and dosage for treatment of reproductive disorders. However, rather than allowing this fact to discourage use of probiotics, the scientific community needs to robustly explore probiotics as potential therapies for improving reproductive health.

#### Biomarkers

Biomarkers are molecular entities with clinical utility in early detection, classification, prognosis, predicting response to therapy, and theranosis of a patient's disease. Specific and sensitive biomarkers can lead to optimal individualized management strategies including active surveillance, expectant management, and surgical or other interventions. They can also lead to new therapeutic trajectories. Biomarkers can reduce over-treatment, under-treatment and incorrect treatment. Understanding the complexity of a microbiome-mediated disease, such as by dysbiosis (microbial imbalance), is critical, as most of these diseases are not determined or defined by a single or even a few changes in the microbial community structure. Capturing the molecular heterogeneity of microbiome-associated disease is the first and necessary step for a successful diagnostics strategy. This, and systematic addition of effective targeted interventions to the treatment options contribute toward the main goal of personalized (genomic) medical diagnostics (point of care predictors), by identification of microbiome quantitative traits or QTLs that predict the risk for a clinical condition.

To achieve the ideal of individualized medicine through understanding the human microbiome, we must understand the symbiotic relationship between humans and microbes by characterizing the microbiome of several mucosal tissues. Adding microbiome profiles to the repertoire of biomarkers for prevention, early detection and diagnosis of primary infections, complications and other health care conditions is crucial. For example, fecal-based microbiome and metabolite signatures could provide unique opportunities for radically changing the detection of chronic inflammation through identification of modifiable dietary risk factors, and through recognition of high-risk populations within whom endoscopic efforts can be focused. These biomarkers could be used to identify populations at risk for disease associated with inflammation as well as to monitor response of diet, probiotic, antibiotic, or other preventive interventions.

## Summary

It is evident based on the expansive literature available to date, that an individual's microbiome serves as more than just a bacterial presence that resides in our body's natural orifices. These microbial environments are composed of numerous communities that function to create a symbiotic host-microbe relationship through regulation of the immune system and healthy tissue physiology. Microbial community dynamics may fluctuate in species diversity and richness but can maintain a “normal” microbial community due to redundancy among specific strains. However, too much fluctuation in any microbial community leaves the host susceptible to not only bacterial over-colonization but also to pathogen entry. In this event there can be serious damage to cellular layers and tissues of a physiological system that may lead to systemic and multiple system impediments, including reproductive efficiency.

In this review we have highlighted literature indicating that communication between the host immune system and the microbial communities that reside in the vaginal cavity impact reproductive health. Further elucidation of host-microbe interactions within the vaginal cavity will enhance our understanding of reproductive disease pathogenesis and potential causes for impaired fertility. Overall, constant and vigilant screening of the human microbiomes will provide early biomarkers in patients that are deviating from a “normal” state prior to becoming clinical symptomatic. More importantly understanding what is and how to achieve the “perfect” balance of the human microbiomes will provide the best medical weapon for individualized personnel medicine for prophylactic treatment of many pathogenic conditions.

### Conflict of interest statement

The authors declare that the research was conducted in the absence of any commercial or financial relationships that could be construed as a potential conflict of interest.

## References

[B1] Aagaard K. Ma J. Antony K. M. Ganu R. Petrosino J. Versalovic J. (2014). The placenta harbors a unique microbiome. Sci. Transl. Med. 6, 237ra265 10.1126/scitranslmed.3008599 PMC4929217 24848255

[B2] Aagaard K. Riehle K. Ma J. Segata N. Mistretta T. A. Coarfa C.. (2012). A metagenomic approach to characterization of the vaginal microbiome signature in pregnancy. PLoS ONE 7:e36466. 10.1371/journal.pone.0036466 22719832 PMC3374618

[B3] Aaltonen J. Ojala T. Laitinen K. Poussa T. Ozanne S. Isolauri E. (2011). Impact of maternal diet during pregnancy and breastfeeding on infant metabolic programming: a prospective randomized controlled study. Eur. J. Clin. Nutr. 65, 10–19. 10.1038/ejcn.2010.225 20948557

[B4] Adlercreutz H. Martin F. Jarvenpaa P. Fotsis T. (1979). Steroid absorption and enterohepatic recycling. Contraception 20, 201–223. 10.1016/0010-7824(79)90094-5 389544

[B5] Antony K. M. Ma J. Mitchell K. B. Racusin D. A. Versalovic J. Aagaard K. (2014). The preterm placental microbiome varies in association with excess maternal gestational weight gain. Am. J. Obstet. Gynecol. [Epub ahead of print]. 10.1016/j.ajog.2014.12.041 25557210 PMC4892181

[B6] Barbonetti A. Cinque B. Vassallo M. R. Mineo S. Francavilla S. Cifone M. G.. (2011). Effect of vaginal probiotic lactobacilli on *in vitro*-induced sperm lipid peroxidation and its impact on sperm motility and viability. Fertil. Steril. 95, 2485–2488. 10.1016/j.fertnstert.2011.03.066 21497805

[B7] Barrington J. W. Linton D. O'Leary A. Blackwell A. Brick J. Calvert J. P. (1997). Anaerobic (bacterial) vaginosis and premalignant disease of the cervix. J. Obstet. Gynaecol. 17, 383–385. 10.1080/01443619750112943 15511897

[B8] Bradshaw C. S. Morton A. N. Hocking J. Garland S. M. Morris M. B. Moss L. M.. (2006). High recurrence rates of bacterial vaginosis over the course of 12 months after oral metronidazole therapy and factors associated with recurrence. J. Infect. Dis. 193, 1478–1486. 10.1086/503780 16652274

[B9] Brocklehurst P. Gordon A. Heatley E. Milan S. J. (2013). Antibiotics for treating bacterial vaginosis in pregnancy. Cochrane Database Syst. Rev. 1:CD000262. 10.1002/14651858.CD000262 23440777 PMC11307253

[B10] Brotman R. M. (2011). Vaginal microbiome and sexually transmitted infections: an epidemiologic perspective. J. Clin. Invest. 121, 4610–4617. 10.1172/JCI57172 22133886 PMC3225992

[B11] Brotman R. M. Erbelding E. J. Jamshidi R. M. Klebanoff M. A. Zenilman J. M. Ghanem K. G. (2007). Findings associated with recurrence of bacterial vaginosis among adolescents attending sexually transmitted diseases clinics. J. Pediatr. Adolesc. Gynecol. 20, 225–231. 10.1016/j.jpag.2006.11.009 17673134 PMC3647449

[B12] Brotman R. M. Klebanoff M. A. Nansel T. R. Yu K. F. Andrews W. W. Zhang J.. (2010). Bacterial vaginosis assessed by gram stain and diminished colonization resistance to incident gonococcal, chlamydial, and trichomonal genital infection. J. Infect. Dis. 202, 1907–1915. 10.1086/657320 21067371 PMC3053135

[B13] Cabrera-Rubio R. Collado M. C. Laitinen K. Salminen S. Isolauri E. Mira A. (2012). The human milk microbiome changes over lactation and is shaped by maternal weight and mode of delivery. Am. J. Clin. Nutr. 96, 544–551. 10.3945/ajcn.112.037382 22836031

[B14] Cai J. L. Qin L. H. Hu Z. Y. (2010). [The development of systematic evolution of ligands by exponential enrichment in tuberculosis]. Zhonghua Jie He He Hu Xi Za Zhi 33, 210–211. 20450642

[B15] Centers for Disease Control Prevention Workowski K. A. Berman S. M. (2006). Sexually transmitted diseases treatment guidelines, 2006. MMWR Recomm. Rep. 55, 1–94. 16888612

[B16] Chung H. Pamp S. J. Hill J. A. Surana N. K. Edelman S. M. Troy E. B.. (2012). Gut immune maturation depends on colonization with a host-specific microbiota. Cell 149, 1578–1593. 10.1016/j.cell.2012.04.037 22726443 PMC3442780

[B17] Cole C. B. Fuller R. Mallet A. K. Rowland I. R. (1985). The influence of the host on expression of intestinal microbial enzyme activities involved in metabolism of foreign compounds. J. Appl. Bacteriol. 59, 549–553. 10.1111/j.1365-2672.1985.tb03359.x 3938453

[B18] Costello E. K. Lauber C. L. Hamady M. Fierer N. Gordon J. I. Knight R. (2009). Bacterial community variation in human body habitats across space and time. Science 326, 1694–1697. 10.1126/science.1177486 19892944 PMC3602444

[B19] Dabek M. McCrae S. I. Stevens V. J. Duncan S. H. Louis P. (2008). Distribution of beta-glucosidase and beta-glucuronidase activity and of beta-glucuronidase gene gus in human colonic bacteria. FEMS Microbiol. Ecol. 66, 487–495. 10.1111/j.1574-6941.2008.00520.x 18537837

[B20] Dechen T. C. Sumit K. Ranabir P. (2010). Correlates of vaginal colonization with group B streptococci among pregnant women. J. Glob. Infect. Dis. 2, 236–241. 10.4103/0974-777X.68536 20927284 PMC2946679

[B21] Decker E. Hornef M. Stockinger S. (2011). Cesarean delivery is associated with celiac disease but not inflammatory bowel disease in children. Gut. Microbes. 2, 91–98. 10.4161/gmic.2.2.15414 21637025

[B22] Denslow S. A. Westreich D. J. Firnhaber C. Michelow P. Williams S. Smith J. S. (2011). Bacterial vaginosis as a risk factor for high-grade cervical lesions and cancer in HIV-seropositive women. Int. J. Gynaecol. Obstet. 114, 273–277. 10.1016/j.ijgo.2011.03.011 21683359

[B23] Dielubanza E. J. Schaeffer A. J. (2011). Urinary tract infections in women. Med. Clin. North Am. 95, 27–41. 10.1016/j.mcna.2010.08.023 21095409

[B24] DiGiulio D. B. (2012). Diversity of microbes in amniotic fluid. Semin. Fetal. Neonatal. Med. 17, 2–11. 10.1016/j.siny.2011.10.001 22137615

[B25] Dominguez F. Galan A. Martin J. J. Remohi J. Pellicer A. Simon C. (2003). Hormonal and embryonic regulation of chemokine receptors CXCR1, CXCR4, CCR5 and CCR2B in the human endometrium and the human blastocyst. Mol. Hum. Reprod. 9, 189–198. 10.1093/molehr/gag024 12651900

[B26] Donders G. (2010). Diagnosis and management of bacterial vaginosis and other types of abnormal vaginal bacterial flora: a review. Obstet. Gynecol. Surv. 65, 462–473. 10.1097/OGX.0b013e3181e09621 20723268

[B27] Donders G. G. Van Bulck B. Van de Walle P. Kaiser R. R. Pohlig G. Gonser S.. (2010). Effect of lyophilized lactobacilli and 0.03 mg estriol (Gynoflor(R)) on vaginitis and vaginosis with disrupted vaginal microflora: a multicenter, randomized, single-blind, active-controlled pilot study. Gynecol. Obstet. Invest. 70, 264–272. 10.1159/000314016 21051846

[B28] Douvier S. Neuwirth C. Filipuzzi L. Kisterman J. P. (1999). Chorioamnionitis with intact membranes caused by Capnocytophaga sputigena. Eur. J. Obstet. Gynecol. Reprod. Biol. 83, 109–112. 10.1016/S0301-2115(98)00240-1 10221619

[B29] Doyle R. M. Alber D. G. Jones H. E. Harris K. Fitzgerald F. Peebles D.. (2014). Term and preterm labour are associated with distinct microbial community structures in placental membranes which are independent of mode of delivery. Placenta 35, 1099–1101. 10.1016/j.placenta.2014.10.007 25458966

[B30] Drell T. Lillsaar T. Tummeleht L. Simm J. Aaspollu A. Vain E.. (2013). Characterization of the vaginal micro- and mycobiome in asymptomatic reproductive-age Estonian women. PLoS ONE 8:e54379. 10.1371/journal.pone.0054379 23372716 PMC3553157

[B31] Egbase P. E. al-Sharhan M. al-Othman S. al-Mutawa M. Udo E. E. Grudzinskas J. G. (1996). Incidence of microbial growth from the tip of the embryo transfer catheter after embryo transfer in relation to clinical pregnancy rate following *in-vitro* fertilization and embryo transfer. Hum. Reprod. 11, 1687–1689. 10.1093/oxfordjournals.humrep.a019470 8921117

[B32] Egbase P. E. Udo E. E. Al-Sharhan M. Grudzinskas J. G. (1999). Prophylactic antibiotics and endocervical microbial inoculation of the endometrium at embryo transfer. Lancet 354, 651–652. 10.1016/S0140-6736(99)02415-0 10466674

[B33] Fanca-Berthon P. Hoebler C. Mouzet E. David A. Michel C. (2010). Intrauterine growth restriction not only modifies the cecocolonic microbiota in neonatal rats but also affects its activity in young adult rats. J. Pediatr. Gastroenterol. Nutr. 51, 402–413. 10.1097/MPG.0b013e3181d75d52 20601908

[B34] Fanchin R. Harmas A. Benaoudia F. Lundkvist U. Olivennes F. Frydman R. (1998). Microbial flora of the cervix assessed at the time of embryo transfer adversely affects in vitro fertilization outcome. Fertil. Steril. 70, 866–870. 10.1016/S0015-0282(98)00277-5 9806568

[B35] Fardini Y. Chung P. Dumm R. Joshi N. Han Y. W. (2010). Transmission of diverse oral bacteria to murine placenta: evidence for the oral microbiome as a potential source of intrauterine infection. Infect. Immun. 78, 1789–1796. 10.1128/IAI.01395-09 20123706 PMC2849412

[B36] Fichorova R. N. Yamamoto H. S. Delaney M. L. Onderdonk A. B. Doncel G. F. (2011). Novel vaginal microflora colonization model providing new insight into microbicide mechanism of action. MBio 2, e00168–e00111. 10.1128/mBio.00168-11 22027006 PMC3202752

[B37] Filicori M. Flamigni C. Meriggiola M. C. Ferrari P. Michelacci L. Campaniello E.. (1991). Endocrine response determines the clinical outcome of pulsatile gonadotropin-releasing hormone ovulation induction in different ovulatory disorders. J. Clin. Endocrinol. Metab. 72, 965–972. 10.1210/jcem-72-5-965 1902487

[B38] Fredericks D. N. Relman D. A. (1996). Sequence-based identification of microbial pathogens: a reconsideration of Koch's postulates. Clin. Microbiol. Rev. 9, 18–33. 8665474 10.1128/cmr.9.1.18 PMC172879

[B39] Fredricks D. N. Fiedler T. L. Marrazzo J. M. (2005). Molecular identification of bacteria associated with bacterial vaginosis. N. Engl. J. Med. 353, 1899–1911. 10.1056/NEJMoa043802 16267321

[B40] Gadelle D. Raibaud P. Sacquet E. (1985). beta-Glucuronidase activities of intestinal bacteria determined both *in vitro* and *in vivo* in gnotobiotic rats. Appl. Environ. Microbiol. 49, 682–685. 3994372 10.1128/aem.49.3.682-685.1985 PMC373571

[B41] Gajer P. Brotman R. M. Bai G. Sakamoto J. Schutte U. M. Zhong X. (2012). Temporal dynamics of the human vaginal microbiota. Sci. Transl. Med. 4, 132ra152 10.1126/scitranslmed.3003605 PMC3722878 22553250

[B42] Gautray J. P. de Brux J. Tajchner G. Robel P. Mouren M. (1981). Clinical investigation of the menstrual cycle. III. Clinical, endometrial, and endocrine aspects of luteal defect. Fertil. Steril. 35, 296–303. 7202753 10.1016/s0015-0282(16)45374-4

[B43] Gillet E. Meys J. F. Verstraelen H. Verhelst R. De Sutter P. Temmerman M.. (2012). Association between bacterial vaginosis and cervical intraepithelial neoplasia: systematic review and meta-analysis. PLoS ONE 7:e45201. 10.1371/journal.pone.0045201 23056195 PMC3462776

[B44] Groeger D. O'Mahony L. Murphy E. F. Bourke J. F. Dinan T. G. Kiely B.. (2013). Bifidobacterium infantis 35624 modulates host inflammatory processes beyond the gut. Gut. Microbes. 4, 325–339. 10.4161/gmic.25487 23842110 PMC3744517

[B45] Gronlund M. M. Gueimonde M. Laitinen K. Kociubinski G. Gronroos T. Salminen S.. (2007). Maternal breast-milk and intestinal bifidobacteria guide the compositional development of the Bifidobacterium microbiota in infants at risk of allergic disease. Clin. Exp. Allergy. 37, 1764–1772. 10.1111/j.1365-2222.2007.02849.x 17941914

[B46] Guarino A. Wudy A. Basile F. Ruberto E. Buccigrossi V. (2012). Composition and roles of intestinal microbiota in children. J. Matern. Fetal. Neonatal. Med. 25(Suppl. 1), 63–66. 10.3109/14767058.2012.663231 22348506

[B47] Guijon F. Paraskevas M. Rand F. Heywood E. Brunham R. McNicol P. (1992). Vaginal microbial flora as a cofactor in the pathogenesis of uterine cervical intraepithelial neoplasia. Int. J. Gynaecol. Obstet. 37, 185–191. 10.1016/0020-7292(92)90379-W 1351005

[B48] Gupta M. McDougal A. Safe S. (1998). Estrogenic and antiestrogenic activities of 16alpha- and 2-hydroxy metabolites of 17beta-estradiol in MCF-7 and T47D human breast cancer cells. J. Steroid. Biochem. Mol. Biol. 67, 413–419. 10.1016/S0960-0760(98)00135-6 10030690

[B49] Han Y. W. Ikegami A. Bissada N. F. Herbst M. Redline R. W. Ashmead G. G. (2006). Transmission of an uncultivated Bergeyella strain from the oral cavity to amniotic fluid in a case of preterm birth. J. Clin. Microbiol. 44, 1475–1483. 10.1128/JCM.44.4.1475-1483.2006 16597879 PMC1448680

[B50] Han Y. W. Redline R. W. Li M. Yin L. Hill G. B. McCormick T. S. (2004). Fusobacterium nucleatum induces premature and term stillbirths in pregnant mice: implication of oral bacteria in preterm birth. Infect. Immun. 72, 2272–2279. 10.1128/IAI.72.4.2272-2279.2004 15039352 PMC375172

[B51] Han Y. W. Shen T. Chung P. Buhimschi I. A. Buhimschi C. S. (2009). Uncultivated bacteria as etiologic agents of intra-amniotic inflammation leading to preterm birth. J. Clin. Microbiol. 47, 38–47. 10.1128/JCM.01206-08 18971361 PMC2620857

[B52] Harris J. Brown H. (1927). Bacterial content of the uterus at cesarean secion. Am. J. Obstet. Gynecol. 13, 133.

[B53] Hickey D. K. Patel M. V. Fahey J. V. Wira C. R. (2011). Innate and adaptive immunity at mucosal surfaces of the female reproductive tract: stratification and integration of immune protection against the transmission of sexually transmitted infections. J. Reprod. Immunol. 88, 185–194. 10.1016/j.jri.2011.01.005 21353708 PMC3094911

[B54] Hickey R. J. Abdo Z. Zhou X. Nemeth K. Hansmann M. Osborn T. W. III. (2013). Effects of tampons and menses on the composition and diversity of vaginal microbial communities over time. BJOG 120, 695–704. 10.1111/1471-0528.12151 23398859

[B55] Hill G. B. (1998). Preterm birth: associations with genital and possibly oral microflora. Ann. Periodontol. 3, 222–232. 10.1902/annals.1998.3.1.222 9722706

[B56] Hill J. A. Anderson D. J. (1992). Human vaginal leukocytes and the effects of vaginal fluid on lymphocyte and macrophage defense functions. Am. J. Obstet. Gynecol. 166, 720–726. 10.1016/0002-9378(92)91703-D 1536258

[B57] Hou D. Zhou X. Zhong X. Settles M. L. Herring J. Wang L.. (2013). Microbiota of the seminal fluid from healthy and infertile men. Fertil. Steril. 100, 1261–1269. 10.1016/j.fertnstert.2013.07.1991 23993888 PMC3888793

[B58] Human Microbiome Jumpstart Reference Strains Consortium, Nelson K. E. Weinstock G. M. Highlander S. K. Worley K. C. Creasy H. H.. (2010). A catalog of reference genomes from the human microbiome. Science 328, 994–999. 10.1126/science.1183605 20489017 PMC2940224

[B59] Human Microbiome Project Consortium (2012a). Structure, function and diversity of the healthy human microbiome. Nature 486, 207–214. 10.1038/nature11234 22699609 PMC3564958

[B60] Human Microbiome Project Consortium. (2012b). A framework for human microbiome research. Nature 486, 215–221. 10.1038/nature11209 22699610 PMC3377744

[B61] Huurre A. Kalliomaki M. Rautava S. Rinne M. Salminen S. Isolauri E. (2008). Mode of delivery - effects on gut microbiota and humoral immunity. Neonatology 93, 236–240. 10.1159/000111102 18025796

[B62] Hyman R. W. Fukushima M. Jiang H. Fung E. Rand L. Johnson B.. (2013). Diversity of the vaginal microbiome correlates with preterm birth. Reprod. Sci. 21, 32–40. 10.1177/1933719113488838 23715799 PMC3857766

[B63] Hyman R. W. Herndon C. N. Jiang H. Palm C. Fukushima M. Bernstein D.. (2012). The dynamics of the vaginal microbiome during infertility therapy with *in vitro* fertilization-embryo transfer. J. Assist. Reprod. Genet. 29, 105–115. 10.1007/s10815-011-9694-6 22222853 PMC3270134

[B64] Jaiyeoba O. Lazenby G. Soper D. E. (2011). Recommendations and rationale for the treatment of pelvic inflammatory disease. Expert. Rev. Anti. Infect. Ther. 9, 61–70. 10.1586/eri.10.156 21171878

[B65] Kabat G. C. Chang C. J. Sparano J. A. Sepkovie D. W. Hu X. P. Khalil A.. (1997). Urinary estrogen metabolites and breast cancer: a case-control study. Cancer Epidemiol. Biomarkers Prev. 6, 505–509. 9232337

[B66] Kaul R. Pettengell C. Sheth P. M. Sunderji S. Biringer A. MacDonald K.. (2008). The genital tract immune milieu: an important determinant of HIV susceptibility and secondary transmission. J. Reprod. Immunol. 77, 32–40. 10.1016/j.jri.2007.02.002 17395270

[B67] Kaushic C. (2009). The role of the local microenvironment in regulating susceptibility and immune responses to sexually transmitted viruses in the female genital tract. J. Reprod. Immunol. 83, 168–172. 10.1016/j.jri.2009.08.010 19857903

[B68] Kim Y. G. Ohta T. Takahashi T. Kushiro A. Nomoto K. Yokokura T.. (2006). Probiotic Lactobacillus casei activates innate immunity via NF-kappaB and p38 MAP kinase signaling pathways. Microbes. Infect. 8, 994–1005. 10.1016/j.micinf.2005.10.019 16513392

[B69] Klebanoff S. J. Coombs R. W. (1991). Viricidal effect of Lactobacillus acidophilus on human immunodeficiency virus type 1: possible role in heterosexual transmission. J. Exp. Med. 174, 289–292. 10.1084/jem.174.1.289 1647436 PMC2118880

[B70] Ley R. E. Knight R. Gordon J. I. (2007). The human microbiome: eliminating the biomedical/environmental dichotomy in microbial ecology. Environ. Microbiol. 9, 3–4. 10.1111/j.1462-2920.2006.01222_3.x 17227400

[B71] Ley R. E. Peterson D. A. Gordon J. I. (2006). Ecological and evolutionary forces shaping microbial diversity in the human intestine. Cell 124, 837–848. 10.1016/j.cell.2006.02.017 16497592

[B72] Ling Z. Liu X. Chen W. Luo Y. Yuan L. Xia Y.. (2013). The restoration of the vaginal microbiota after treatment for bacterial vaginosis with metronidazole or probiotics. Microb. Ecol. 65, 773–780. 10.1007/s00248-012-0154-3 23250116

[B73] Liversedge N. H. Turner A. Horner P. J. Keay S. D. Jenkins J. M. Hull M. G. (1999). The influence of bacterial vaginosis on *in-vitro* fertilization and embryo implantation during assisted reproduction treatment. Hum. Reprod. 14, 2411–2415. 10.1093/humrep/14.9.2411 10469722

[B74] Lozupone C. A. Stombaugh J. I. Gordon J. I. Jansson J. K. Knight R. (2012). Diversity, stability and resilience of the human gut microbiota. Nature 489, 220–230. 10.1038/nature11550 22972295 PMC3577372

[B75] Luthje P. Hirschberg A. L. Brauner A. (2014). Estrogenic action on innate defense mechanisms in the urinary tract. Maturitas 77, 32–36. 10.1016/j.maturitas.2013.10.018 24296328

[B76] Ma B. Forney L. J. Ravel J. (2012). Vaginal microbiome: rethinking health and disease. Annu. Rev. Microbiol. 66, 371–389. 10.1146/annurev-micro-092611-150157 22746335 PMC3780402

[B77] Makino H. Kushiro A. Ishikawa E. Kubota H. Gawad A. Sakai T.. (2013). Mother-to-infant transmission of intestinal bifidobacterial strains has an impact on the early development of vaginally delivered infant's microbiota. PLoS ONE 8:e78331. 10.1371/journal.pone.0078331 24244304 PMC3828338

[B78] Mandar R. (2013). Microbiota of male genital tract: impact on the health of man and his partner. Pharmacol. Res. 69, 32–41. 10.1016/j.phrs.2012.10.019 23142212

[B79] Manichanh C. Varela E. Martinez C. Antolin M. Llopis M. Dore J.. (2008). The gut microbiota predispose to the pathophysiology of acute postradiotherapy diarrhea. Am. J. Gastroenterol. 103, 1754–1761. 10.1111/j.1572-0241.2008.01868.x 18564125

[B80] Mardis E. R. (2008). Next-generation DNA sequencing methods. Annu. Rev. Genomics Hum. Genet. 9, 387–402. 10.1146/annurev.genom.9.081307.164359 18576944

[B81] Martin H. L. Richardson B. A. Nyange P. M. Lavreys L. Hillier S. L. Chohan B.. (1999). Vaginal lactobacilli, microbial flora, and risk of human immunodeficiency virus type 1 and sexually transmitted disease acquisition. J. Infect. Dis. 180, 1863–1868. 10.1086/315127 10558942

[B82] Mendz G. L. Kaakoush N. O. Quinlivan J. A. (2013). Bacterial aetiological agents of intra-amniotic infections and preterm birth in pregnant women. Front. Cell Infect. Microbiol. 3:58. 10.3389/fcimb.2013.00058 24137568 PMC3797391

[B83] Mirmonsef P. Gilbert D. Zariffard M. R. Hamaker B. R. Kaur A. Landay A. L.. (2011). The effects of commensal bacteria on innate immune responses in the female genital tract. Am. J. Reprod. Immunol. 65, 190–195. 10.1111/j.1600-0897.2010.00943.x 21143335 PMC3581076

[B84] Ness R. B. Hillier S. L. Richter H. E. Soper D. E. Stamm C. McGregor J.. (2002). Douching in relation to bacterial vaginosis, lactobacilli, and facultative bacteria in the vagina. Obstet. Gynecol. 100, 765. 10.1016/S0029-7844(02)02184-1 12383547

[B85] Nicol A. F. Fernandes A. T. Bonecini-Almei da Mda G. (2005). Immune response in cervical dysplasia induced by human papillomavirus: the influence of human immunodeficiency virus-1 co-infection – review. Mem. Inst. Oswaldo. Cruz. 100, 1–12. 10.1590/S0074-02762005000100001 15867955

[B86] Obst J. M. Seamark R. F. (1975). Hormone studies on ewes grazing an oestrogenic (Yarloop clover) pasture during the reproductive cycle. Aust. J. Biol. Sci. 28, 279–290. 1180769 10.1071/bi9750279

[B87] Parkin D. M. (2006). The global health burden of infection-associated cancers in the year 2002. Int. J. Cancer 118, 3030–3044. 10.1002/ijc.21731 16404738

[B88] Payne M. S. Bayatibojakhi S. (2014). Exploring preterm birth as a polymicrobial disease: an overview of the uterine microbiome. Front. Immunol. 5:595. 10.3389/fimmu.2014.00595 25505898 PMC4245917

[B89] Petricevic L. Domig K. J. Nierscher F. J. Sandhofer M. J. Fidesser M. Krondorfer I.. (2014). Characterisation of the vaginal Lactobacillus microbiota associated with preterm delivery. Sci. Rep. 4, 5136. 10.1038/srep05136 24875844 PMC4038809

[B90] Platz-Christensen J. J. Sundstrom E. Larsson P. G. (1994). Bacterial vaginosis and cervical intraepithelial neoplasia. Acta Obstet. Gynecol. Scand. 73, 586–588. 10.3109/00016349409006278 8079612

[B91] Plottel C. S. Blaser M. J. (2011). Microbiome and malignancy. Cell Host. Microbe. 10, 324–335. 10.1016/j.chom.2011.10.003 22018233 PMC3264051

[B92] Quan M. (2010). Vaginitis: diagnosis and management. Postgrad. Med. 122, 117–127. 10.3810/pgm.2010.11.2229 21084788

[B93] Ravel J. Gajer P. Abdo Z. Schneider G. M. Koenig S. S. McCulle S. L.. (2011). Vaginal microbiome of reproductive-age women. Proc. Natl. Acad. Sci. U.S.A. 108, 4680–4687. 10.1073/pnas.1002611107 20534435 PMC3063603

[B94] Reid G. Burton J. Devillard E. (2004). The rationale for probiotics in female urogenital healthcare. MedGenMed 6, 49. 15208560 PMC1140735

[B95] Reid J. N. Bisanz J. E. Monachese M. Burton J. P. Reid G. (2013). The rationale for probiotics improving reproductive health and pregnancy outcome. Am. J. Reprod. Immunol. 69, 558–566. 10.1111/aji.12086 23414386

[B96] Rifkin S. B. Smith M. R. Brotman R. M. Gindi R. M. Erbelding E. J. (2009). Hormonal contraception and risk of bacterial vaginosis diagnosis in an observational study of women attending STD clinics in Baltimore, MD. Contraception 80, 63–67. 10.1016/j.contraception.2009.01.008 19501217

[B97] Roger L. C. Costabile A. Holland D. T. Hoyles L. McCartney A. L. (2010). Examination of faecal Bifidobacterium populations in breast- and formula-fed infants during the first 18 months of life. Microbiology 156, 3329–3341. 10.1099/mic.0.043224-0 20864478

[B98] Round J. L. Mazmanian S. K. (2009). The gut microbiota shapes intestinal immune responses during health and disease. Nat. Rev. Immunol. 9, 313–323. 10.1038/nri2515 19343057 PMC4095778

[B99] Sartor R. B. (2004). Therapeutic manipulation of the enteric microflora in inflammatory bowel diseases: antibiotics, probiotics, and prebiotics. Gastroenterology 126, 1620–1633. 10.1053/j.gastro.2004.03.024 15168372

[B100] Satokari R. Gronroos T. Laitinen K. Salminen S. Isolauri E. (2009). Bifidobacterium and Lactobacillus DNA in the human placenta. Lett. Appl. Microbiol. 48, 8–12. 10.1111/j.1472-765X.2008.02475.x 19018955

[B101] Sha B. E. Zariffard M. R. Wang Q. J. Chen H. Y. Bremer J. Cohen M. H.. (2005). Female genital-tract HIV load correlates inversely with Lactobacillus species but positively with bacterial vaginosis and Mycoplasma hominis. J. Infect. Dis. 191, 25–32. 10.1086/426394 15592999

[B102] Sirota I. Zarek S. M. Segars J. H. (2014). Potential influence of the microbiome on infertility and assisted reproductive technology. Semin. Reprod. Med. 32, 35–42. 10.1055/s-0033-1361821 24390919 PMC4137456

[B103] Taylor B. D. Darville T. Haggerty C. L. (2013). Does bacterial vaginosis cause pelvic inflammatory disease? Sex Transm. Dis. 40, 117–122. 10.1097/OLQ.0b013e31827c5a5b 23324974

[B104] Tojo R. Suarez A. Clemente M. G. de los Reyes-Gavilan C. G. Margolles A. Gueimonde M.. (2014). Intestinal microbiota in health and disease: role of bifidobacteria in gut homeostasis. World J. Gastroenterol. 20 15163–15176. 10.3748/wjg.v20.i41.15163 25386066 PMC4223251

[B105] Tringe S. G. von Mering C. Kobayashi A. Salamov A. A. Chen K. Chang H. W.. (2005). Comparative metagenomics of microbial communities. Science 308, 554–557. 10.1126/science.1107851 15845853

[B106] van Oostrum N. De Sutter P. Meys J. Verstraelen H. (2013). Risks associated with bacterial vaginosis in infertility patients: a systematic review and meta-analysis. Hum. Reprod. 28, 1809–1815. 10.1093/humrep/det096 23543384

[B107] Vassallo M. F. Walker W. A. (2008). Neonatal microbial flora and disease outcome. Nestle Nutr. Workshop Ser. Pediatr. Program. 61, 211–224. 10.1159/000113496 18196954

[B108] Vitali B. Cruciani F. Baldassarre M. E. Capursi T. Spisni E. Valerii M. C.. (2012). Dietary supplementation with probiotics during late pregnancy: outcome on vaginal microbiota and cytokine secretion. BMC Microbiol. 12:236. 10.1186/1471-2180-12-236 23078375 PMC3493352

[B109] Walther-Antonio M. R. Jeraldo P. Berg Miller M. E. Yeoman C. J. Nelson K. E. Wilson B. A.. (2014). Pregnancy's stronghold on the vaginal microbiome. PLoS ONE 9:e98514. 10.1371/journal.pone.0098514 24896831 PMC4045671

[B110] Witkin S. S. Kligman I. Grifo J. A. Rosenwaks Z. (1995). Chlamydia trachomatis detected by polymerase chain reaction in cervices of culture-negative women correlates with adverse *in vitro* fertilization outcome. J. Infect. Dis. 171, 1657–1659. 10.1093/infdis/171.6.1657 7769313

[B111] Yarbrough V. L. Winkle S. Herbst-Kralovetz M. M. (2014). Antimicrobial peptides in the female reproductive tract: a critical component of the mucosal immune barrier with physiological and clinical implications. Hum. Reprod. Update. [Epub ahead of print]. 10.1093/humupd/dmu065 25547201

[B112] Yeoman C. J. Thomas S. M. Miller M. E. Ulanov A. V. Torralba M. Lucas S.. (2013). A multi-omic systems-based approach reveals metabolic markers of bacterial vaginosis and insight into the disease. PLoS ONE 8:e56111. 10.1371/journal.pone.0056111 23405259 PMC3566083

